# Vaginal dysbiosis and inflammatory signatures in preterm labor: an integrated model for predicting preterm birth

**DOI:** 10.3389/fimmu.2026.1809046

**Published:** 2026-06-05

**Authors:** Subeen Hong, Gi Soo Um, Byung Soo Kang, Oyoung Kim, Seon Ui Lee, Hyun Sun Ko, Sangho Nam, Seungok Lee, In Yang Park, Sun Shin

**Affiliations:** 1 Department of Obstetrics and Gynecology, Seoul St. Mary’s Hospital, College of Medicine, The Catholic University of Korea, Seoul, Republic of Korea; 2 Department of Obstetrics and Gynecology, Yeouido St. Mary’s Hospital, College of Medicine, The Catholic University of Korea, Seoul, Republic of Korea; 3 Department of Obstetrics and Gynecology, Incheon St. Mary’s Hospital, College of Medicine, The Catholic University of Korea, Seoul, Republic of Korea; 4 Department of Microbiology, College of Medicine, The Catholic University of Korea, Seoul, Republic of Korea; 5 Department of Medical Sciences, Graduate School of The Catholic University of Korea, Seoul, Republic of Korea; 6 Department of Laboratory Medicine, College of Medicine, The Catholic University of Korea, Seoul, Republic of Korea

**Keywords:** biomarkers, dysbiosis, matrix metalloproteinase, microbiota, pregnancy, premature birth

## Abstract

**Background:**

Preterm birth (PTB) is a major cause of neonatal morbidity and mortality, with vaginal microbiome dysbiosis and local immune responses implicated in its pathogenesis. However, the role of vaginal immune and extracellular matrix (ECM) remodeling factors in the progression from preterm labor (PTL) to PTB remains unclear. This study examines the associations between microbiome composition and immune and ECM-related protein composition in PTL patients to identify key factors and predictive models associated with the risk of PTB.

**Methods:**

This prospective study included 136 pregnant women classified into three groups: Control (Term Birth, TB), PTL-TB (Preterm Labor with Term Birth), and PTL-PTB (Preterm Labor with Preterm Birth). Vaginal microbiome composition was analyzed using 16S rRNA sequencing and categorized by dysbiosis status and community state types (CST). Cytokines (IL-1β, IFN-γ, IL-2, IL-4, IL-6, IL-8, IL-10, IL-12p70) and ECM remodeling enzymes (MMP-1, MMP-2, MMP-3, MMP-7, MMP-8, MMP-9, MMP-12, MMP-13, IGFBP-1) were quantified in vaginal secretions using the Luminex^®^ Assay multiplex kit. A multivariable logistic regression model was constructed to predict preterm birth, using significant microbiome, cytokine, and MMP variables, with performance evaluated by ROC analysis.

**Results:**

Dysbiosis and CST IV were more prevalent in the PTL-PTB group. IL-1β was highest in CST III, while MMP-9 and other MMPs were elevated in CST IV. CVF MMP-9 was consistently increased across PTL-PTB cases, dysbiosis, and CST IV. However, IGFBP-1, MMP-8, and MMP-13 were significantly different by clinical outcome but not correlated with microbiome composition. A logistic regression model incorporating non-Lactobacillus fraction, IGFBP-1, MMP-9, MMP-13, and TNF-α demonstrated excellent predictive performance (AUC = 0.910) for PTB.

**Conclusions:**

Distinct microbial and immune profiles are associated with the progression from PTL to PTB. MMP-9 may serve as a key effector linking dysbiosis to extracellular matrix remodeling and PTB. Integrative biomarker models may support early risk stratification in women with PTL.

## 1 Introduction

Preterm birth (PTB) is a leading cause of neonatal morbidity and mortality, with an estimated global prevalence of approximately 10% (1). One of the major causes of PTB is spontaneous preterm labor (PTL), accounting for around 50% of all cases (2). Intra-amniotic infection and inflammation are considered key contributors to spontaneous PTL, and are hypothesized to result from the ascending spread of pathogenic vaginal microbiota (3, 4).

Over the past decade, the expansion of microbiome research has led to numerous studies reporting an association between vaginal dysbiosis and PTB. These studies consistently suggest that a high relative abundance of *Lactobacillus crispatus* in the vaginal microbiota confers a protective effect against PTB, and such findings have been replicated across Asian, European, and other global populations (5, 6).

In addition, the vaginal microbiome has been classified into community state types (CSTs), and numerous studies have investigated the relationships between microbial composition and host immune responses, including their underlying pathogenic mechanisms. These studies have consistently reported that Lactobacillus-dominant vaginal microbiota is generally associated with lower levels of proinflammatory cytokines, whereas non-*Lactobacillus*-dominant communities, such as CST IV, are linked to increased vaginal inflammation and epithelial barrier disruption through the upregulation of inflammatory cytokines and matrix metalloproteinases (MMPs) (7–10).

However, most existing studies have compared PTB with term birth (TB), and there is a paucity of information that comprehensively analyzes the vaginal microbiome, inflammatory cytokines, and MMPs in relation to the progression from PTL to PTB. A deeper understanding of how microbial and immune responses associated with PTL are linked to the actual occurrence of PTB is clinically important. Furthermore, the simultaneous analysis of clinical characteristics, microbiota composition, cytokines, and MMPs from the same cervicovaginal sample is essential for accurately interpreting these complex interactions.

We hypothesized that preterm birth is associated with distinct alterations in vaginal microbial composition and host inflammatory responses, and that integrated host–microbiome profiling may provide insight into the pathophysiological mechanisms underlying PTL and PTB. Therefore, in this study, we aimed to investigate the vaginal microbiome, inflammatory cytokines, and MMPs associated with PTL and PTB, in order to identify host–microbiome features that distinguish preterm from term birth and support clinical risk stratification.

## 2 Materials and methods

### 2.1 Study design and population

This study was a prospective comparative study conducted at Seoul St. Mary’s Hospital from October 2020 to August 2023. The study population consisted of women presenting with PTL and healthy pregnant controls without a history of PTL. Women with PTL were subsequently categorized into two subgroups according to delivery outcome: those who delivered preterm before 37 weeks of gestation (PTL-PTB group) and those who ultimately delivered at term (PTL-TB group). Healthy pregnant women without PTL who were enrolled during routine outpatient prenatal visits served as the control group. A total of 136 women were included in the study, comprising 70 women with PTL and 66 women without PTL (control group). Among those with PTL, 26 delivered preterm (PTL-PTB group) and 44 delivered at term (PTL-TB group). This study aimed to investigate the microbiome composition and its associated cytokine profiles in relation to PTL and PTB, comparing these findings across the three groups.

The inclusion criteria consisted of singleton pregnancies, maternal age between 19 and 50 years, and a gestational age of at least 18 weeks. Exclusion criteria included preterm premature rupture of membranes (PPROM) before sample collection, multifetal pregnancies, medically indicated preterm births, loss to follow-up, and insufficient or improperly collected samples.

All eligible participants received a detailed explanation of the study, and written informed consent was obtained before enrollment. This study was approved by the Institutional Review Board (IRB) of Seoul St. Mary’s Hospital (IRB KC21TISI0621) and was conducted in accordance with the Declaration of Helsinki.

### 2.2 Data and sample collection

Upon patient enrollment, samples were collected prior to the administration of any medication, and maternal baseline characteristics and ultrasound findings were recorded. For sample collection, a flocked swab (NFS-2 swab, Noble Biosciences, Inc., Hwaseong-si, Korea) was gently applied to the posterior fornix of vaginal wall. The collected cervicovaginal fluid (CVF) sample was then suspended in 1 mL of phosphate-buffered saline (PBS) and immediately transported to the laboratory, where it was stored at −80 °C until further analysis.

### 2.3 Definition and management of PTL

PTL was defined as the presence of regular uterine contractions necessitating hospitalization and medical intervention. PTB was defined as delivery occurring before 37 weeks of gestation. At our institution, PTL management involved tocolytic therapy and selective administration of prophylactic antibiotics, which were only used when clinical chorioamnionitis or other infections were suspected. Additionally, corticosteroids were administered between 24 and 34 weeks of gestation to enhance fetal lung development.

### 2.4 16s rRNA gene sequencing

Genomic DNA was extracted from vaginal samples using the FastDNA Spin kit (MP biomedicals, Irvine, CA, USA). The V3-V4 regions of the 16S rRNA gene were amplified using primers 341F (5’-AATGATACGGCGACCACCGAGATCTACAC-XXXXXXXX-TCGTCGGCAGCGTC-AGATGTGTATAAGAGACAG-CCTACGGGNGGCWGCAG-3’; underlined sequence indicates the target region primer) and 805R (5’- CAAGCAGAAGACGGCATACGAGAT-XXXXXXXX-GTCTCGTGGGCTCGG-AGATGTGTATAAGAGACAG-GACTACHVGGGTATCTAATCC-3’). Polymerase Chain Reaction (PCR) was performed using Herculase II Fusion DNA Polymerase (Agilent Technologies, Santa Clara, CA, USA) with the Nextera XT Index Kit v2 (Illumina, San Diego, CA, USA) under the following conditions: 95 °C for 3 min; 25 cycles of 95 °C for 30 s, 55 °C for 30 s, and 72 °C for 30 s; and a final extension at 72 °C for 5 min. Amplicons were verified by agarose gel electrophoresis, purified with CleanPCR (CleanNA, Waddinxveen, Netherlands), and pooled in equimolar concentrations. Library quality was assessed on a 2100 Bioanalyzer (Agilent Technologies), and sequencing was performed on an Illumina MiSeq platform at CJ Bioscience, Inc. and Macrogen, Inc. (Seoul, Republic of Korea).

### 2.5 Bioinformatics analyses

Raw sequence quality was assessed using FastQC (v0.12.1) (https://www.bioinformatics.babraham.ac.uk/projects/fastqc/). Reads were trimmed using Trimmomatic (v0.39) (24695404) with a sliding window (Q15,4 bp). Reads shorter than 36 bp after trimming were discarded. Adapters and primers were removed with Cutadapt (v5.0) (DOI: 10.14806/ej.17.1.200). Subsequently, reads were filtered to exclude those with a median quality score below Q20 or containing ambiguous bases. Paired reads were merged using PANDAseq (v2.11) (22333067), requiring a minimum overlap of 30 bp.

The resulting demultiplexd reads were processed using the QIIME 2 pipeline (v2024.02) (31341288). Taxonomy was assigned to the generated amplicon sequence variants (ASVs) using a naïve-Bayes classifier trained on the SILVA 138.1 SSURef NR99 database (23193283). To improve taxonomic resolution, assignments were cross-referenced with NCBI GenBank resources for the STIRRUPS database (23282177), and key classifications were manually validated using NCBI BLAST. For downstream analysis, ASVs present in fewer than two samples or with a total relative abundance of <0.01% were excluded. Additionally, suspected contaminant ASVs, characterized by a consistent proportional abundance within experimental batches, were manually removed. Finally, vaginal CSTs were determined using the VALENCIA classifier (33228810), categorizing samples into CST I (*L. crispatus*-dominated), CST II (*L. gasseri*-dominated), CST III (*L. iners*-dominated), CST IV (polymicrobial community with depleted lactobacilli), and CST V (*L. jensenii*-dominated).

Alpha diversity and beta diversity were assessed by microeco R package (v1.11.0) (40770112) based on Shannon entropy and Pielou’s evenness, and principal coordinate analysis (PCoA) based on Bray-Curtis distance, respectively. Co-occurrence networks were constructed using the R package NetCoMi (v1.2.0) (33264391) based on Spearman correlations, and node sizes were scaled by Eigenvector centrality. Only pathways with >5% contribution from a single species across all samples and adjusted *p*-value < 0.1 were retained.

### 2.6 Quantification of vaginal inflammatory proteins, and MMPs in CVF

CVF cytokines and matrix metalloproteinases (MMPs), including C–C motif chemokine ligand (CCL) 4/Macrophage inflammatory protein 1-beta (MIP-1β), Interferon gamma (IFN-γ), insulin-like growth factor binding protein-1 (IGFBP-1), Interleukin (IL)-1β, IL-2, IL-4, IL-6, IL-8, IL-10, IL-12p70, MMP-1, MMP-2, MMP-3, MMP-7, MMP-8, MMP-9, MMP-12, MMP-13, and Tumor necrosis factor alpha (TNF-α), were measured using a 19-plex Human Luminex^®^ Discovery Assay (LXSAHM-19, R&D Systems, Minneapolis, MN, USA) according to the manufacturer’s protocol.

Samples were thawed and centrifuged at 10,000 × g for 2 min, and the supernatant was analyzed without dilution. The reaction between the sample and bead-antibody mixture was performed at room temperature for 2 hours, following the standard protocol.

The fluorescence intensity (Median Fluorescence Intensity, MFI) was detected using a Luminex^®^ system, and cytokine concentrations were calculated using MasterPlex QT 2010 software (MiraiBio, Hitachi, CA, USA). Standard curves were generated by the best-fit method based on the five-parameter logistic (5-PL) regression model, and analyte concentrations were interpolated accordingly.

### 2.7 Statistical analysis

Differences in continuous variables across the three clinical groups were assessed using the Kruskal-Wallis test, followed by Dunn’s test for multiple comparisons with Bonferroni correction. The relationship between the relative abundance of *Lactobacillus* and the concentrations of individual cytokines was investigated using simple linear regression. The strength and significance of these associations were evaluated based on the coefficient of determination (R²) and the *p*-value. All statistical tests were two-tailed, and a *p*-value < 0.05 was considered statistically significant. To identify significant predictors of PTB, univariate logistic regression analyses were performed for each candidate biomarker, comparing the PTL-PTB with the control group. Variables with a significant univariate association were included in a multivariate model. The diagnostic accuracy was evaluated using Receiver Operating Characteristic (ROC) curve analysis and the Area Under the Curve (AUC).

To further validate the predictive power of the selected biomarkers using a machine learning approach, a Random Forest (RF) classification model was also applied using the caret R package. The dataset was randomly partitioned into a training set (60% of the samples) and a testing set (40%). A fixed random seed was applied during data partitioning and model training to ensure reproducibility. Using the train () function, the RF model was then trained exclusively on the training data via a 5-fold cross-validation (CV) procedure to tune hyperparameters and prevent overfitting. The model was constructed using the same five predictor variables and was built with 1000 trees (ntree), while the optimal number of variables to consider at each split (mtry) was determined through the CV process. The performance of the trained model was assessed in the testing dataset by calculating the AUC using the pROC R package.

## 3 Results

### 3.1 Clinical characteristics of study participants

Patient characteristics and delivery outcomes are summarized in **Table 1**. There were no significant differences in maternal age or body mass index (BMI) among the three groups. However, a history of previous preterm birth was significantly more frequent in the PTL-PTB group (15.4%) compared to the PTL-TB (4.5%) and control (1.5%) groups (p = 0.03). The gestational age at sampling was significantly earlier in the PTL groups (29.9 weeks in PTL-PTB, 30.7 weeks in PTL-TB) than in the control group (37.0 weeks, p < 0.001). Regarding delivery outcomes, the gestational age at delivery was significantly lower in the PTL-PTB group (34.3 weeks) compared to both the PTL-TB (38.2 weeks) and control (39.0 weeks) groups (p < 0.001).

**Table 1:** Clinical characteristics and delivery outcomes of study participants.

Variables	PTL-PTB (n=26)	PTL-TB (n=44)	Control (n=66)	p-value
Maternal characteristics				
Maternal age (years)	33.0 (32.5-37.7)	35.0 (32.2-36.0)	33.0 (31.0-35.2)	0.104
Prepregnancy BMI	20.7 (20.1-24.0)	20.7 (19.7-22.4)	21.0 (19.4-23.9)	0.663
History of preterm birth	4 (15.4%)	2 (4.5%)	1 (1.5%)	0.030
Gestational age at sampling (weeks)	29.9 (26.3-32.2)	30.7 (27.2-32.6)	37.0 (36.3-37.2)	<0.001
Cervical length at sampling (cm)	2.1 (0.8-2.9)	2.3 (1.8-3.1)	2.8 (2.2-3.4)	0.004
Positive vaginal culture	9(34.6%)	15 (34.1%)	24 (36.4%)	0.971
Delivery outcomes				
Gestational age at delivery (weeks)	34.3 (33.0-35.5)	38.2 (37.3-38.6)	39.0 (37.6-39.5)	<0.001
Cesarean section	22 (84.6%)	27 (61.4%)	27 (40.9%)	<0.001
Birthweight (kg)	2.3 (2.1-2.5)	3.1 (2.9-3.4)	3.1 (2.8-3.4)	<0.001

Data are presented as median (interquartile range) or n (%). p-values were calculated using the Kruskal–Wallis test for continuous variables and chi-square or Fisher’s exact test for categorical variables, as appropriate. BMI, body mass index; PTL, preterm labor; PTB, preterm birth; TB, term birth.

### 3.2 Vaginal microbiome profiles associated with PTL and PTB

We obtained 4,710,869 high-quality sequence reads from 136 samples, with an average of 34,639 reads per sample (range: 1,149 to 133,041). They were clustered into 678 unique amplicon sequence variants (ASVs), all of which were successfully assigned a taxonomic classification.

**Figure 1:**
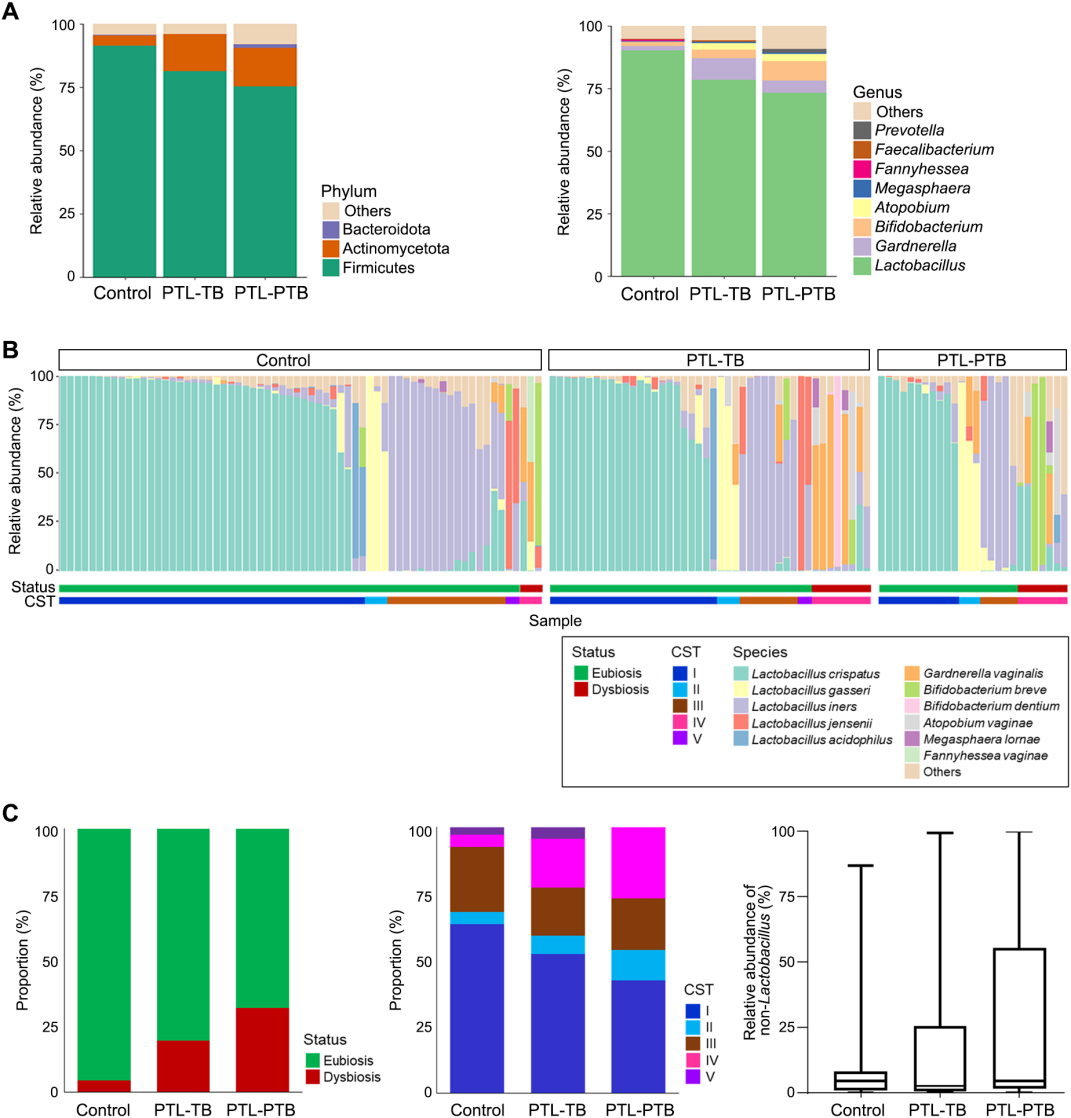
Vaginal microbiome profiles associated with preterm birth. **(A)** Bar plots showing the average relative abundance of vaginal microbiota at the phylum (left) and genus (right) levels across the Control, preterm labor with term birth (PTL-TB), and preterm labor with preterm birth (PTL-PTB) groups. **(B)** Stacked bar plot displaying the species-level composition for each individual sample. The annotation bars below the main plot indicate the assigned dysbiosis status (Eubiosis or Dysbiosis) and Community State Type (CST) for each sample. **(C)** Quantitative assessment of the vaginal microbial health status across the clinical groups. The plots display the proportion of dysbiosis (left), the distribution of CSTs (middle), and the relative abundance of non-*Lactobacillus* genera (right). .

**Figure 1** presents the distinct microbial composition profiles across the groups. At the phylum level, all groups were dominated by *Firmicutes*, with a higher abundance of *Actinomycetota* in the PTL groups. These differences were more pronounced at the genus level, where the PTL groups, particularly the PTL-PTB group, showed a decrease in the relative abundance of *Lactobacillus* and a corresponding increase in anaerobic genera such as *Gardnerella*, *Bifidobacterium*, *Atopobium*, and *Prevotella* (**Figure 1A**). The control group was characterized by eubiosis, with communities dominated by Lactobacillus species (*L. crispatus*, *L. iners*, *L. jensenii*) corresponding to CSTs I, III, and V. In contrast, the PTL-PTB group was defined by dysbiosis, featuring CST IV. The PTL-TB group exhibited an intermediate profile with a mixture of these eubiotic and dysbiotic community types (**Figure 1B**).

Accordingly, the proportion of samples classified as dysbiotic was minimal in the control group but increased to approximately 25% in the PTL-TB group and over 75% in the PTL-PTB group. The distribution of CSTs shows a clear shift from *Lactobacillus*-dominant types (CST I, II, III, V) in the control group to a community overwhelmingly dominated by CST IV in the PTL-PTB group (**Figure 1C**).

### 3.3 Microbial diversity and co-occurrence networks

**Figure 2:**
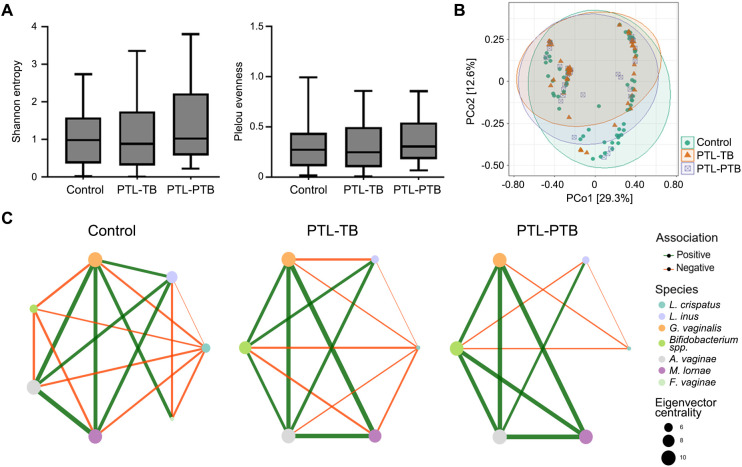
Microbial diversity and co-occurrence networks of the vaginal microbiome. **(A)** Box plots showing alpha diversity metrics, Shannon entropy (left) and Pielou’s evenness (right), for the Control, preterm labor with term birth (PTL-TB), and preterm labor with preterm birth (PTL-PTB) groups. **(B)** Beta diversity analysis visualized by a Principal Coordinate Analysis (PCoA) plot based on Bray-Curtis dissimilarity. Each point represents a single sample, colored by its respective group. Ellipses represent the 95% confidence interval for each group. The percentage of variation explained by each principal coordinate is shown on the axes. **(C)** Microbial co-occurrence networks for each clinical group. Nodes represent key bacterial species, with their size indicating their influence within the network (eigenvector centrality). Edges represent significant correlations, with green lines indicating a positive association and orange lines indicating a negative association. The thickness of the line corresponds to the strength of the correlation.

**Figure 2** shows microbial diversity and network analysis across study groups. Alpha diversity analysis showed a higher microbial diversity in the PTL-PTB group compared to the control and PTL-TB groups, although these differences were not statistically significant (**Figure 2A**). Beta diversity analysis showed the difference in the overall microbial composition among three groups. Specifically, the control group forms a relatively distinct cluster from the two PTL groups, and the PTL-TB and PTL-PTB groups largely overlap, indicating a similar compositional structure between women experiencing PTL, regardless of their ultimate delivery outcome (ANOSIM R = 0.061, P = 0.020) (**Figure 2B**).

Microbial co-occurrence network analysis revealed distinct interaction patterns between the groups (**Figure 2C**). In the control group, *L. crispatus* played a central role and showed strong negative correlations with bacterial vaginosis (BV)-associated bacteria like *G. vaginalis* and *A. vaginae*, suggesting an inhibitory or competitive relationship. In contrast, the network structure was completely reversed in the PTL-PTB group. A strong, positively correlated network formed among BV-associated bacteria (*G. vaginalis*, *A. vaginae*, *F. vaginae*), suggesting a synergistic polymicrobial community associated with PTB risk.

### 3.4 Inflammatory proteins and MMPs in CVF in relation to microbiome status and PTB

**Figure 3:**
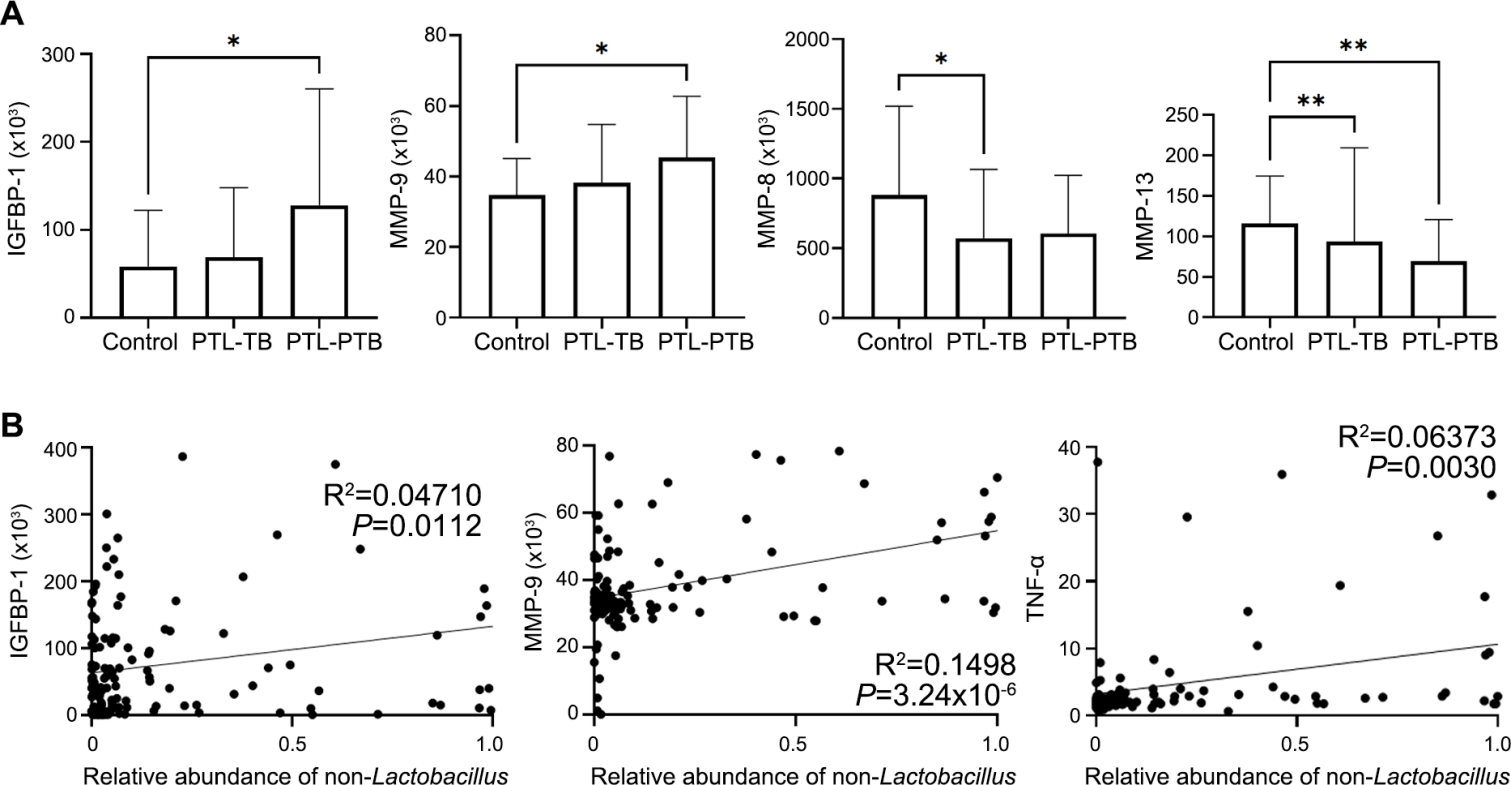
Vaginal inflammatory proteins and their association with microbiome composition. **(A)** Bar plots comparing the vaginal fluid levels of IGFBP-1, MMP-9, MMP-8, and MMP-13 across the three clinical groups: Control, preterm labor with term birth (PTL-TB), and preterm labor with preterm birth (PTL-PTB). Data are presented as mean ± standard deviation. Asterisks indicate statistically significant differences between groups (*p < 0.05, **p < 0.01). **(B)** Scatter plots illustrating the positive correlation between the relative abundance of non-*Lactobacillus* taxa and the levels of IGFBP-1, MMP-9, and TNF-α. The line represents the linear regression fit. R² and P-values from the correlation analysis are shown for each plot. All analyte concentrations are expressed in picograms per milliliter (pg/mL).

**Figure 3** and **Supplementary Table 1** summarizes the distribution of inflammatory cytokines, chemokines and MMPs among the three groups. Among the analytes, IGFBP-1 (p = 0.035), MMP-13 (p = 0.001), MMP-8 (p = 0.013), and MMP-9 (p = 0.020) showed statistically significant differences (**Figure 3A**). IGFBP-1 and MMP-9 levels were highest in the PTL-PTB group (p = 0.035 and p = 0.020, respectively), whereas MMP-13 and MMP-8 were lowest (p = 0.001 and p = 0.013, respectively). However, *post hoc* analysis revealed no significant differences between the PTL-PTB and PTL-TB groups. Compared to controls, the PTL-PTB group showed higher levels of IGFBP-1 and MMP-9 and lower levels of MMP-13. The PTL-TB group had significantly lower MMP-8 and MMP-13 levels than the control group.

Correlation analysis between inflammatory markers and microbiome composition supported these findings (**Figure 3B**). There were significant positive correlations between the relative abundance of non-*Lactobacillus* taxa and several inflammatory markers, including IGFBP-1 (R² = 0.047, p = 0.0112), MMP-9 (R² = 0.15, p < 0.0001), and TNF-α (R² = 0.064, p = 0.0030).

In addition, cytokine and MMP levels were analyzed by microbiome classification (**Supplementary Table 2**). In the overall cohort, MMP-9, MMP-2, MMP-7, and TNF-α levels were significantly elevated in both the dysbiosis group and the CST IV subgroup, whereas IL-1β was most highly expressed in CST III. Notably, MMP-9 was consistently elevated across clinical phenotypes, microbiome status, and CST classifications, suggesting its potential as a robust indicator of PTB risk.

### 3.5 Predictive modeling for PTB risk

**Figure 4:**
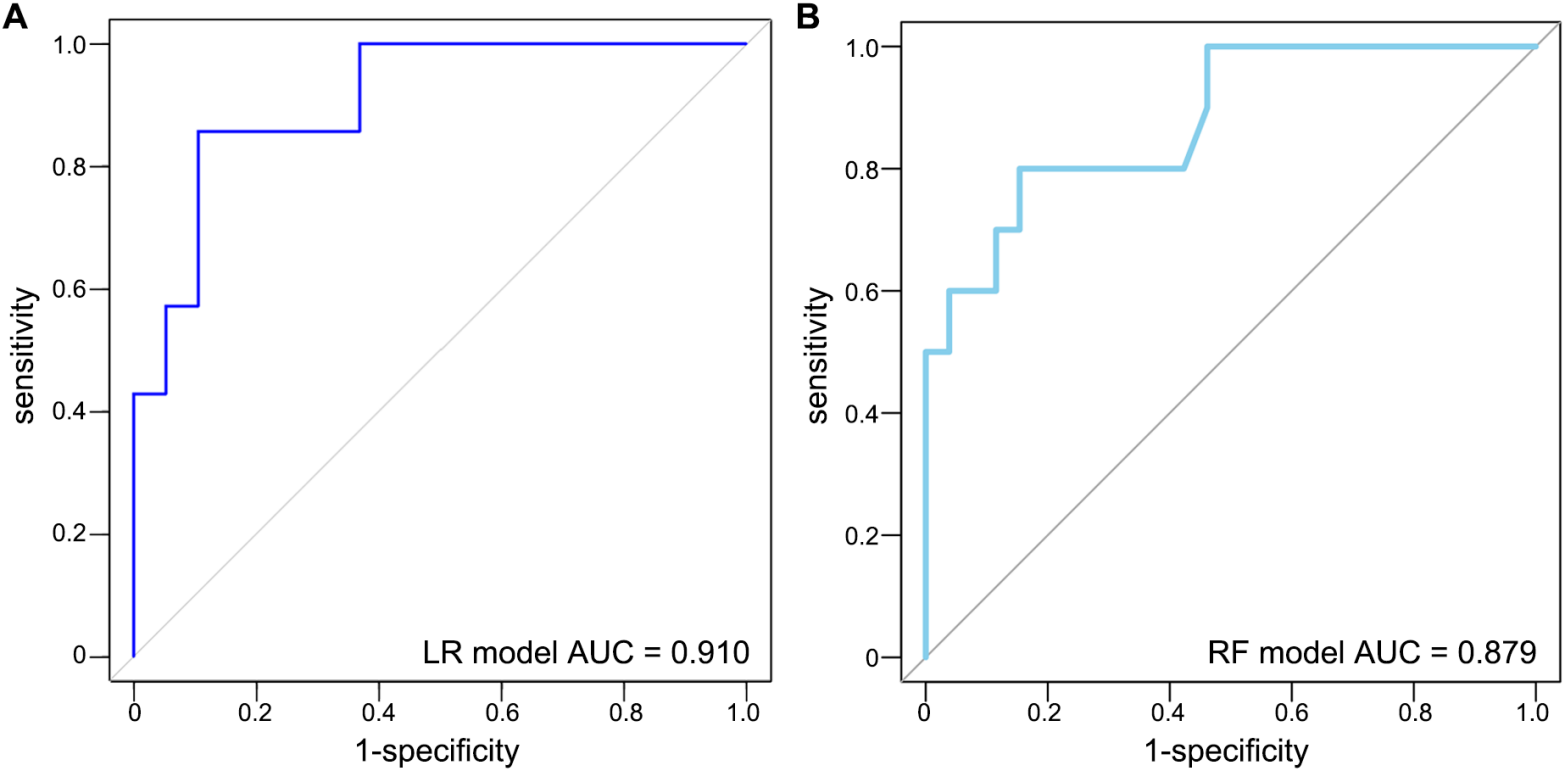
Predictive performance of models for preterm birth risk. **(A)** Receiver Operating Characteristic (ROC) curves for the multivariable Logistic Regression (LR) model, achieving an Area Under the Curve (AUC) of 0.910. **(B)** ROC curve for the Random Forest (RF) model, achieving an AUC of 0.879.

Based on the variables significantly associated with preterm birth in univariate analysis (**Supplementary Table 3**), we first constructed a multivariable logistic regression model. This model included five key variables: the relative abundance of non-*Lactobacillus*, IGFBP-1, MMP-9, MMP-13, and TNF-α. The logistic regression model demonstrated excellent predictive performance for PTB, achieving an AUC of 0.910 (**Figure 4A**).

To further validate the predictive power of these biomarkers using a machine learning approach, we developed a Random Forest model with the same set of five variables. The model was trained and optimized using a 5-fold cross-validation scheme to identify the best-performing hyperparameter (mtry). The performance of the optimized Random Forest model was robust, achieving an accuracy of 83.3% (95% CI: 67.2% – 93.6%). The model yielded a sensitivity of 80.0%, a specificity of 84.6%, and an overall AUC of 0.879 (**Figure 4B**). Collectively, the strong performance of both the logistic regression and Random Forest models highlights the potential of combining specific host immune markers and microbiome-derived metrics to accurately stratify the risk of PTB.

## 4 Discussion

Among the various inflammatory markers evaluated, MMP-9 consistently demonstrated strong associations with vaginal dysbiosis, CST IV dominance, and PTL with PTB outcomes. In addition, we developed a multivariable prediction model incorporating MMP-9 along with IGFBP-1, MMP-13, TNF-α, and the relative abundance of non-Lactobacillus taxa, which achieved excellent performance (AUC = 0.910) in stratifying PTB risk. These findings suggest that MMP-9 may serve not only as a key effector linking vaginal microbiome disruption to extracellular matrix remodeling processes leading to PTB, but also as a clinically useful biomarker when integrated into a composite prediction model.

MMP-9, a member of the MMP family, plays a key role in extracellular matrix degradation, particularly in the chorion, amnion, and cervical stroma. Its involvement in tissue remodeling during labor and parturition has made it a candidate biomarker for PTB. Several studies have investigated associations between PTB and MMP-9 concentrations in amniotic fluid (AF), maternal blood, and CVF (11–13). In a study by Hong et al., no significant correlation was observed between AF and CVF MMP-9 levels, and CVF MMP-9 demonstrated lower predictive value for intra-amniotic infection or imminent delivery compared to AF MMP-9 (13). However, contrasting results have been reported in several prospective studies, where elevated vaginal MMP-9 concentrations were associated with cervical ripening, and early MMP-9 elevation was linked to an increased risk of preterm birth (14–16). In our study, MMP-9 was not only elevated in the PTL-PTB group but also showed strong correlations with CST IV, vaginal dysbiosis, and non-Lactobacillus taxa abundance. This convergence across clinical, microbial, and immune parameters reinforces its potential as both a mechanistic effector and a practical biomarker of PTB.

This CVF elevation of MMP-9 related to PTB and vaginal dysbiosis may be explained by several biological mechanisms. Unlike amniotic fluid or systemic circulation, the cervicovaginal space is in direct contact with the vaginal mucosa and cervical stroma, which are both rich in epithelial and immune cells capable of producing MMP-9 in response to local microbial or inflammatory stimuli. For example, *Gardnerella vaginalis* has been shown to activate Toll-like receptor 2 signaling pathways, leading to the secretion of MMP-9 (16, 17). In addition, proinflammatory proteins, which are often elevated in CST IV-dominated microbial communities, may in turn stimulate the production of MMP-9 at the mucosal surface (18–20). These local interactions between the microbiome, epithelial immunity, and extracellular matrix remodeling create a uniquely responsive environment in the CVF, making it a sensitive compartment for detecting MMP-9–mediated pathways involved in preterm birth.

The positive correlations between non-Lactobacillus abundance and IGFBP-1, MMP-9, and TNF-α further support the presence of a host–microbiome interaction associated with PTB. *Lactobacillus*-dominant vaginal microbiota is generally considered to maintain a protective low-inflammatory environment, whereas *Lactobacillus* depletion has been associated with vaginal dysbiosis and adverse pregnancy outcomes (3, 6). In the present study, increased non-*Lactobacillus* abundance was associated with elevated TNF-α and MMP-9 levels, suggesting a link between microbial dysbiosis, inflammatory activation, and extracellular matrix degradation. As a canonical pro-inflammatory cytokine, TNF-α is closely associated with innate immune activation and the downstream pro-inflammatory cytokine cascade. Therefore, these findings suggest that loss of *Lactobacillus* dominance may promote a pro-inflammatory vaginal microenvironment. In addition, the positive association with IGFBP-1 may reflect disruption of the maternal–fetal interface and membrane instability. Collectively, these findings suggest that vaginal dysbiosis may contribute to PTB through coordinated inflammatory and tissue-remodeling pathways involving both microbial and host responses.

One of findings of our study is IL-1β levels were highest in CST III, whereas several MMPs, including MMP-9, were elevated in CST IV. This pattern was also observed in correlation analyses, further supporting the differential inflammatory profiles associated with distinct CSTs. *Lactobacillus iners* has been repeatedly associated with increased proinflammatory cytokines in prior studies. Several reports have shown that IL-1β levels are significantly elevated in CST III (20–22), consistent with our findings. One recent study also reported a positive correlation between IL-1β and the absolute abundance of L. iners (20), suggesting that this species may contribute to a host-mediated inflammatory response, possibly by inducing macrophage colony-stimulating factor (M-CSF) and creating an environment conducive to the growth of opportunistic pathogens (22, 23). This may reflect a transition from cytokine-dominated immune activation in CST III to protease-driven tissue remodeling in CST IV.

Although there is paucity of information about Other MMPs excepting MMP-9 associated with vaginal microbiome, an experimental study conducting by Chene et al. reported elevation MMP-10 and MMP-13 in response to BV and enhanced tissue degrading enzyme activity (24). These findings suggest that MMPs beyond MMP-9 may also be involved in extracellular matrix remodeling in the context of CST IV and dysbiotic vaginal environments.

Although IGFBP-1, MMP-8, and MMP-13 showed significant differences between clinical subgroups, no clear associations were observed with vaginal microbiome composition. Interestingly, even lower levels of MMP-8 and MMP-13 were observed in patients who experienced PTB. One possible explanation is that these markers may be primarily regulated by intrauterine or systemic tissue environments, rather than being directly influenced by the local vaginal microbiota. Alternatively, the temporal dynamics of microbial shifts and protein expression may not coincide, limiting the ability to detect cross-sectional correlations. Indeed, modulation by endocrine factors and tissue-derived inhibitors may underlie observed discrepancies in local versus systemic protease levels (25, 26).

In addition to the descriptive findings, our study constructed a multivariable logistic regression model to predict the risk of PTB following PTL. This model incorporated five variables—non-Lactobacillus fraction, IGFBP-1, MMP-9, MMP-13, and TNF-α—which were selected based on their consistent associations with both microbiome composition and clinical outcome. The model demonstrated excellent predictive performance, achieving an AUC of 0.910, suggesting its potential clinical utility in risk stratification among women with symptomatic PTL. By integrating host immune markers and microbial features into a single predictive framework, this model underscores the importance of a systems-level approach to preterm birth risk assessment. However, external validation in independent cohorts will be essential to confirm its generalizability and clinical applicability.

Strengths of this study include the prospective collection of CVF samples under a standardized protocol, which ensured consistency in sampling and biomarker analysis. Unlike many previous studies that grouped all causes of PTB together, our study focused exclusively on PTL, allowing for a more clinically relevant interpretation. Furthermore, by stratifying patients into clinically meaningful PTL subtypes, we were able to examine differential risk patterns. Importantly, this study provides novel scientific insights by integrating clinical context, vaginal microbiome composition, and CVF levels of inflammatory cytokines and MMPs, all analyzed from the same sample source.

Limitations of our study include the use of single timepoint sampling, which limited our ability to evaluate dynamic changes over time. In addition, because healthy controls were sampled at later gestational ages than the PTL groups, gestational age-related differences in the vaginal microbiome and immune milieu cannot be completely excluded. Although microbiome profiling was conducted using validated methods, we were unable to employ shotgun metagenomic sequencing or other advanced high-resolution approaches, which may have allowed for a more precise characterization of microbial composition and function. Moreover, the relatively modest sample size of this exploratory prospective cohort may have limited statistical power for detecting smaller differences in certain inflammatory mediators. Therefore, some potentially relevant immune signatures may not have been fully identified in the current study, and larger validation studies with longitudinal approaches are warranted.

In conclusion, we identified distinct immune-microbial patterns associated with PTL and PTB. Vaginal dysbiosis and CST IV were more prevalent in preterm cases, and MMP-9 emerged as a consistently elevated marker across clinical, microbial, and immune phenotypes. While cytokine differences were less pronounced, our integrative model combining microbiota composition, immune, growth factor, and MMPs achieved high predictive performance (AUC = 0.910). These findings highlight the potential of cervicovaginal biomarkers in understanding PTB mechanisms and improving early risk stratification in symptomatic pregnancies.

## Data Availability

The datasets generated and/or analyzed during the current study are not publicly available due to ethical and privacy restrictions related to patient clinical data (as approved by the Institutional Review Board). However, anonymized datasets can be made available from the corresponding author upon reasonable request.
