# Supplementary material for: Vaginal dysbiosis and inflammatory signatures in preterm labor: an integrated model for predicting preterm birth
Source: Front Immunol. 2026 Jun 5;17:1809046. doi: 10.3389/fimmu.2026.1809046 (PMC13278985; doi:10.3389/fimmu.2026.1809046)
Supplement: Supplementary file 1 [file SupplementaryFile1.docx]

***Supplementary Information***

**Supplementary Table 1. Distribution of inflammatory cytokines and MMPs among the three study groups**

| analytes (pg/ml) | PTL-PTB  (n=26) | PTL-TB  (n=44) | Control  (n=66) | p-value |
| --- | --- | --- | --- | --- |
| IL-1β | 827.8 (85.7-1617.1) | 333.4 (31.6-1,346.9) | 209.3 (55.1-1,103.3) | 0.349 |
| IL-2 | 42.2 (25.1-92.8) | 31.7 (15.2-76.0) | 36.0 (20.8-67.1) | 0.295 |
| IL-4 | 40.5 (23.4-50.0) | 34.1 (14.0-43.4) | 32.2 (24.5-43.6) | 0.655 |
| IL-6 | 21.4 (12.1-51.2) | 18.1 (10.9-24.1) | 21.8 (17.3-36.9) | 0.155 |
| IL-10 | 0.2 (0.1-0.9) | 0.2 (0.1-0.8) | 0.5 (0.2-0.9) | 0.241 |
| IL-12p70 | 17.0 (3.0-47.0) | 3.0 (3.0-41.2) | 26.0 (3.0-41.2) | 0.329 |
| CCL4/MIP-1β | 238.5 (142.7-401.0) | 166.0 (114.6-268.6) | 170.2 (139.7-242.6) | 0.272 |
| IL-8/CXCL8 | 2,361.3 (627.6-7,180.5) | 1,306.4 (338.1-5,595.3) | 1,179.7 (481.3-3,388.7) | 0.221 |
| TNF-α | 2.4 (1.8-3.1) | 2.2 (1.8-3.3) | 2.2 (1.8-2.8) | 0.409 |
| IFN-γ | 11.6 (7.5-15.23) | 10.1 (5.8-15.0) | 11.1 (8.6-14.2) | 0.708 |
| IGFBP-1 (x 10^3^) | 57.5 (32.5-208.4) | 41.2 (9.5-83.6) | 39.5 (7.5-99.4) | 0.035^*^ |
| MMP-1 | 7.3 (1.4-53.9) | 3.5 (0.2-24.4) | 8.2 (2.8-19.2) | 0.438 |
| MMP-2 | 326.0 (235.9-585.7) | 276.0 (167.1-418.4) | 343.2 (274.1-455.4) | 0.197 |
| MMP-3 | 106.7 (67.8-187.9) | 94.0 (66.1-126.0) | 105.5 (84.8-132.3) | 0.308 |
| MMP-7 | 548.1 (381.6-2359.3) | 418.6 (193.1-1,017.3) | 575.0 (424.6-839.6) | 0.234 |
| MMP-8 (x 10^3^) | 614.9 (307.3-731.9) | 576.6 (93.4-753.9) | 737.3 (527.9-129.3) | 0.013^†^ |
| MMP-9 (x 10^3^) | 37.2 (33.3-56.1) | 34.0 (31.7-46.7) | 33.3 (30.7-35.8) | 0.020^*^ |
| MMP-12 | 19.2 (9.8-30.9) | 17.2 (8.1-35.0) | 21.2 (11.8-28.0) | 0.797 |
| MMP-13 | 70.3 (28.4-116.8) | 72.0 (32.0-121.6) | 111.0 (86.0-139.6) | 0.001^*†^ |

Data are presented as median (interquartile range). p-values were calculated using the Kruskal–Wallis test , followed by the Bonferroni post-hoc test for pairwise comparisons. ^*^Statistically significant difference between PTL-PTB and Control (p < 0.05). ^†^Statistically significant difference between PTL-TB and Control (p < 0.05). MMP, matrix metalloproteinase; PTL, preterm labor; PTB, preterm birth; TB, term birth; IL, interleukin; CCL4, C–C motif chemokine ligand 4; MIP-1β, macrophage inflammatory protein-1 beta; CXCL8, C–X–C motif chemokine ligand 8; TNF-α, tumor necrosis factor-alpha; IFN-γ, interferon gamma; IGFBP‑1, insulin-like growth factor binding protein-1.

**Supplementary Table 2. Distribution of inflammatory cytokines and MMPs by vaginal microbiome classification**

| analytes (pg/ml) | Normal  (n=119) | Dysbiosis  (n=17) | p-value | CST I  (n=76) | CST II  (n=9) | CST III  (n=29) | CST IV  (n=18) | CST V  (n=4) | p-value |
| --- | --- | --- | --- | --- | --- | --- | --- | --- | --- |
| IL-1β | 220.3  (44.8-1271.1) | 724.8  (304.6-1,332.0) | 0.065 | 165.3  (33.7-857.4) | 674.6  (80.8-1501.7) | 1,102.8  (71.1-3,636.0) | 795.0  (334.5-1,654.7) | 254.7  (79.7-478.9) | 0.010 |
| IL-2 | 34.9  (17.3-71.5) | 53.5  (23.2-81.2) | 0.221 | 31.7  (15.0-67.6) | 36.7  (19.9-68.8) | 37.5  (22.9-90.9) | 54.3  (24.3-81.0) | 23.9  (17.2-36.7) | 0.246 |
| IL-4 | 34.0  (22.4-43.7) | 40.7  (28.6-46.0) | 0.264 | 32.3  (22.3-41.3) | 34.1  (26.6-58.5) | 37.5  (26.6-52.3) | 40.5  (29.9-45.3) | 19.1  (9.2-30.1) | 0.233 |
| IL-6 | 20.2  (14.1-31.7) | 23.0  (19.7-54.4) | 0.092 | 20.5  (14.0-29.3) | 23.4  (18.7-83.6) | 19.4  (11.0-60.6) | 22.5  (18.9-52.0) | 18.3  (14.0-20.4) | 0.349 |
| IL-10 | 0.3 (0.2-0.9) | 0.3 (0.2-0.9) | 0.716 | 0.2 (0.1-0.9) | 0.6 (0.3-1.8) | 0.3 (0.2-0.9) | 0.2 (0.2-0.9) | 0.3 (0.1-0.5) | 0.320 |
| IL-12p70 | 20.7  (3.0-41.2) | 27.7  (3.0-41.2) | 0.860 | 20.7  (3.0-41.2) | 27.7  (3.0-58.7) | 20.7  (3.0-47.8) | 31.1  (3.0-41.2) | 3.0  (3.0-9.2) | 0.573 |
| CCL4/MIP-1β | 168.5  (128.0-251.3) | 237.1  (156.2-294.7) | 0.166 | 161.6  (122.8-245.6) | 189.1  (148.7-216.6) | 183.0  (139.4-347.6) | 236.6  (162.9-293.1) | 149.2  (120.6-162.4) | 0.355 |
| IL-8/CXCL8 | 1,241.3  (409.2-4,311.4) | 3,057.6  (769.3-6,735.6) | 0.205 | 1,226.6  (367.2-3,428.7) | 1,664.8  (424.3-2,754.0) | 1,381.8  (565.0-7,760.2) | 2,400.9  (817.2-6,365.9) | 720.3  (573.8-1,642.5) | 0.310 |
| TNF-α | 2.2 (1.8-2.9) | 2.9 (2.2-9.4) | 0.009 | 2.2 (1.8-2.6) | 2.3 (2.0-3.9) | 2.1 (1.8-3.1) | 2.9 (2.2-9.3) | 1.9 (1.8-2.2) | 0.084 |
| IFN-γ | 10.1 (7.2-14.2) | 11.8 (10.1-17.0) | 0.327 | 9.7 (7.2-13.2) | 12.9 (8.6-15.6) | 12.9 (8.6-18.6) | 11.8 (10.1-16.5) | 8.2 (4.1-11.5) | 0.095 |
| IGFBP-1  (x 10^3^) | 43.4  (11.5-104.0) | 38.2  (10.7-164.0) | 0.619 | 40.3  (7.4-106.7) | 54.9  (15.2-57.0) | 46.2  (13.3-114.9) | 39.2  (11.8-159.7) | 51.4  (27.7-71.3) | 0.860 |
| MMP-1 | 7.1 (1.3-19.5) | 19.9 (4.2-51.7) | 0.152 | 4.6 (1.3-17.4) | 18.1 (1.3-42.6) | 14.3 (5.0-23.9) | 17.5 (4.6-48.9) | 2.4 (0.1-5.4) | 0.100 |
| MMP-2 | 324.2  (188.2-434.2) | 441.0  (343.2-676.6) | 0.028 | 324.2  (175.2-398.9) | 414.3  (247.7-570.1) | 348.4  (271.2-583.7) | 433.6  (347.7-650.8) | 224.8  (165.5-260.5) | 0.034 |
| MMP-3 | 101.4  (71.7-131.2) | 116.8  (93.1-166.6) | 0.199 | 98.4  (72.1-117.4) | 113.8  (92.5-180.0) | 132.5  (75.7-212.1) | 111.1  (93.3-162.2) | 68.1  (60.4-74.3) | 0.017 |
| MMP-7 | 531.9  (336.8-691.3) | 1,185.6  (591.1-3449.4) | 0.003 | 525.0  (324.9-651.4) | 578.5  (482.7-722.8) | 545.4  (272.8-1,460.4) | 1,073.7  (583.9-3,418.2) | 511.5  (280.0-663.1) | 0.068 |
| MMP-8  (x 10^3^) | 660.4  (346.6-954.2) | 603.0  (202.8-821.7) | 0.521 | 659.0  (473.9-1,036.8) | 666.3  (289.8-741.9) | 611.4  (338.3-874.7) | 610.7  (242.0-874.7) | 748.4  (550.8-782.6) | 0.990 |
| MMP-9  (x 10^3^) | 33.4  (31.0-37.9) | 52.0  (33.7-58.8) | 0.022 | 33.1  (31.1-35.8) | 31.8  (30.5-37.5) | 37.9  (33.4-48.4) | 44.9  (32.3-58.4) | 34.1  (28.9-36.9) | 0.018 |
| MMP-12 | 17.2 (9.8-29.2) | 21.3 (8.1-33.6) | 0.582 | 17.2 (9.8-28.8) | 28.0 (12.6-30.9) | 17.2 (11.2-23.9) | 21.3 (9.8-33.6) | 5.2 (4.0-10.1) | 0.205 |
| MMP-13 | 99.1  (53.6-130.1) | 72.0  (53.4-108.3) | 0.273 | 103.1  (57.3-130.1) | 120.4  (111.0-134.9) | 85.8  (41.0-118.8) | 68.4  (50.7-107.0) | 75.3  (61.0-92.1) | 0.168 |

Data are presented as median (interquartile range). p-values were calculated using the Mann–Whitney U test (eubiotic vs dysbiotic) and Kruskal–Wallis test (CST subgroups). MMP, matrix metalloproteinase; CST, community state type; IL, interleukin; CCL4, C–C motif chemokine ligand 4; MIP-1β, macrophage inflammatory protein-1 beta; CXCL8, C–X–C motif chemokine ligand 8; TNF-α, tumor necrosis factor-alpha; IFN-γ, interferon gamma; IGFBP‑1, insulin-like growth factor binding protein-1.

**Supplementary Table 3. Univariate logistic regression for key vaginal microbiome features and inflammatory cytokines and MMPs with preterm birth**

| Variables | OR | 95% CI | p-value |
| --- | --- | --- | --- |
| Non-*Lactobacillus* (%) | 14.6 | 2.3-118.6 | 0.006 |
| *G. vaginalis* (%) | 40.7 | 0.3-7661.3 | 0.132 |
| *Bifidobacterium* spp (%) | 6.8 | 0.5-214.0 | 0.164 |
| *A. vaginae* (%) | 2.7x10^18^ | 57.4-2.0x10^74^ | 0.224 |
| *M. lornae* (%) | 2.6 x10^6^ | 0-1.8x10^26^ | 0.314 |
| *F. vaginae* (%) | 0 | NA-4.7x10^277^ | 0.992 |
| IL-1β | 1.1 | 1.0-1.3 | 0.174 |
| IL-2 | 1.2 | 0.8-1.7 | 0.475 |
| IL-4 | 0.9 | 0.6-1.4 | 0.656 |
| IL-6 | 1.1 | 0.8-1.5 | 0.471 |
| IL-10 | 0.8 | 0.3-1.9 | 0.664 |
| IL-12p70 | 0.9 | 0.7-1.2 | 0.492 |
| CCL4/MIP-1β | 1.6 | 1.0-2.6 | 0.086 |
| IL-8/CXCL8 | 1.2 | 0.9-1.5 | 0.159 |
| TNF-α | 1.8 | 1.1-3.3 | 0.033 |
| IFN-γ | 1.0 | 0.6-1.8 | 0.956 |
| IGFBP-1 | 1.4 | 1.1-1.7 | 0.014 |
| MMP-1 | 1.1 | 0.9-1.3 | 0.333 |
| MMP-2 | 1.2 | 0.8-1.8 | 0.459 |
| MMP-3 | 1.2 | 0.8-1.8 | 0.450 |
| MMP-7 | 1.1 | 0.8-1.4 | 0.637 |
| MMP-8 | 0.9 | 0.8-1.1 | 0.361 |
| MMP-9 | 6.4 | 2.1-23.2 | 0.002 |
| MMP-12 | 1.0 | 0.6-1.5 | 0.860 |
| MMP-13 | 0.6 | 0.4-0.8 | 0.002 |

p-values were calculated using the Mann–Whitney U test (Control vs PTL-PTB). OR, odds ratio; CI, confidence interval; MMP, matrix metalloproteinase; CST, community state type; IL, interleukin; CCL4, C–C motif chemokine ligand 4; MIP-1β, macrophage inflammatory protein-1 beta; CXCL8, C–X–C motif chemokine ligand 8; TNF-α, tumor necrosis factor-alpha; IFN-γ, interferon gamma; IGFBP‑1, insulin-like growth factor binding protein-1.
